# 

**Published:** 1883-04-20

**Authors:** 


Entered at the Post-Office at Atlanta, as second-class matter.

VOL. XIII. APRIL 20, 1883. NO. 4.

### THE SOUTHERN MEDICAL RECORD:

### A MONTHLY JOURNAL OF PRACTICAL MEDICINE.

Terms: $2.00 per Annum, in Advance; 4 Copies $6.00; Single Copies 20 Cents.

*"QUICQUID PRÆCIPIES ESTO BREVIS."*

